# Clinical Quiz—Newborn Female with an Anorectal Malformation and a Gynecological Abnormality

**DOI:** 10.1055/s-0041-1741508

**Published:** 2022-04-13

**Authors:** Anisha Apte, Allison Mayhew, Elise McKenna, Veronica Gomez-Lobo, Marc A. Levitt

**Affiliations:** 1 Department of General Surgery, The George Washington University School of Medicine and Health Sciences, Washington, District of Columbia, United States; 2 Department of Pediatric and Adolescent Gynecology, Children's National Medical Center, Washington, District of Columbia, United States; 3 Department of General and Thoracic Surgery, Children's National Medical Center, Washington, District of Columbia, United States; 4 Eunice Kennedy Shriver National Institute of Child Health and Human Development, National Institutes of Health, Bethesda, Maryland, United States

**Keywords:** anorectal malformation, imperforate anus, Mullerian anomalies, vestibular fistula, vaginal atresia

## Abstract

We present a case of a newborn female with imperforate anus who on exam was found to have a rectal fistula in the vestibule, no vaginal opening, and a normal urethra. A diagnostic laparoscopy was performed to elucidate the internal anatomy. The case is presented with a focus on surgical strategies in approaching the female patient with anorectal malformation and a Mullerian anomaly, with questions for the readers posed in a quiz format.

## Case Report

**Fig. 1:**
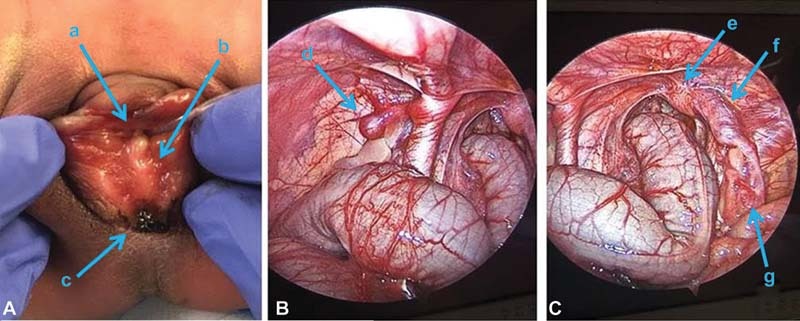
( **A** ) Physical exam showing meconium passing through posterior portion of introitus, with no vaginal orifice in location expected. ( **B** ) Diagnostic laparoscopy showing a left atretic fallopian tube with intact ovary. ( **C** ) Diagnostic laparoscopy showing an intact right fallopian tube with intact ovary. a. Urethra. b. Introitus. c. Rectum. d. Left atretic Mullerian system. e. Right obstructed vagina. f. Right patent uterus. g. Right patent fallopian tube.

A full-term newborn has an imperforate anus, thought to be a rectovestibular fistula. The baby was taken to the operating room on the first day of life with the plan for primary repair of the rectal anomaly via a posterior sagittal anorectoplasty (PSARP). A more careful physical exam in the operating room, however, identified an unexpected finding. This prompted the decision to proceed with a diagnostic laparoscopy instead of definitive surgery. Images from the physical exam and the diagnostic laparoscopy are shown in Fig. 1A .

## Questions

1. What is notable about the physical exam (see Fig. 1A )?
  - The child does not have a urethral opening.
  - This child has a cloacal anomaly.
  - Meconium is passing through the posterior vulvar fourchette and there is no vaginal opening.
  - The vagina is anteriorly located.
2. Based on the perineal exam and the laparoscopic photos (see Fig. 1 ), how would you describe this anorectal malformation (ARM)?
  - Rectovestibular fistula.
  - Rectovaginal fistula.
  - Rectovestibular fistula with distal vaginal atresia.
  - Cloacal anomaly.
3. What would your plan be?
  - PSARP only.
  - PSARP and colovaginoplasty.
  - PSARP and vaginal pull-through.
  - Leave distal rectum as neovagina and pull-through of proximal rectum.
  - PSARP with plan for vaginal dilation in adulthood.

## Discussion

Newborn females with an ARM may present with several anatomic variations. These include perineal fistula, rectovestibular fistula, isolated imperforate anus without fistula, or cloaca. 1 A thorough physical exam in the first 24 hours of life is crucial to identify which anatomic variant of ARM is present. The passing of meconium through a location outside of the sphincter complex signals the presence of a fistula.

In the case presented, examination of the patient revealed meconium passing from an orifice appearing to be within the introitus (see Fig. 1A ). While this was initially presumed to be a typical rectovestibular fistula, with the opening of the rectal fistula abutting the hymen but within the introitus, a more careful examination in the operating room demonstrated the presence of a rectal fistula within the posterior fourchette of the vulva and the absence of a vaginal opening.

**Fig. 2:**
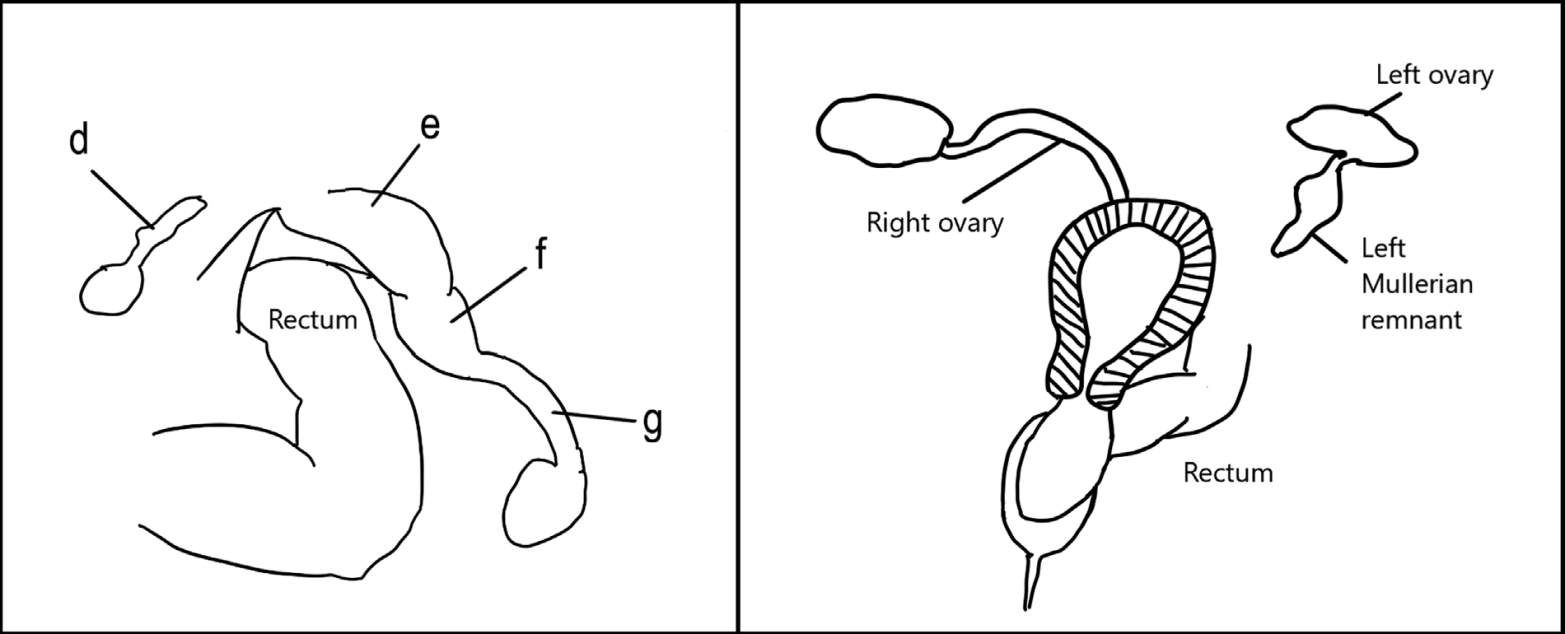
*Left Image.* A schematic drawing of Fig. 1 , with the perspective of looking into the pelvis from a cephalad (bottom border) to caudal (top border) perspective. *Right Image* . Inverted diagram showing cephalad (top border) to caudal (bottom border) orientation. d. Left atretic Mullerian system. e. Right obstructed vagina. f. Right patent uterus. g. Right patent fallopian tube.

After discussion with the family, the decision was made to proceed with a diagnostic laparoscopy to better understand the anatomy and plan for future intervention. Diagnostic laparoscopy revealed an atretic left Mullerian system with the presence of a truncated left fallopian tube and no discernable left uterine, cervical, or vaginal structures, and what appeared to be a developed right Mullerian system including right uterine horn, palpable cervix, and a mildly distended vaginal canal (see Fig. 1A–C ). Together, the external examination and laparoscopic findings indicated the presence of distal vaginal atresia on the right with atretic structures on the left (see Fig. 2 ).

While rectovestibular fistulas are the most common variant of ARM in newborn females, 1 the combination of distal vaginal atresia and rectovestibular fistula is a rare occurrence. It is important for surgeons to be aware of this defect as it is often misdiagnosed and incorrectly treated. 2 The presence of distal vaginal atresia in these cases often goes unrecognized, as the orifice through which meconium passes is presumed to be the typical rectovestibular fistula and the lack of an introitus is not recognized. 2 Sometimes confusion arises when the surgeon attempts a primary repair with PSARP, identifies the rectum, and is then unable to find the vagina. But more often, the vaginal anomaly is not noted and only discovered at puberty when the patient presents with amenorrhea or cyclic pelvic pain. Others may mistake the rectal fistula for the vagina, which leads to difficulty in identifying the rectum. 2

In any of these situations, dissection of the space between the rectum and urethra can create scar tissue that will make any subsequent reconstruction more challenging. 2 This makes identification of this anomaly prior to any surgical repair extremely important. All options for gynecologic intervention need to be considered, including the possibility of a vaginal pull-through if an upper vagina is present, need for vaginal replacement if appropriate, or the potential for future dilations of the introitus if there are no intraabdominal Mullerian structures (similar to the internal anatomy of a patient with Mayer-Rokitansky-Kuster-Hauser (MRKH) syndrome).

On the initial physical exam, it is prudent for the physician to identify both the number of orifices and their location relative to proper anatomic landmarks. 1 3 In both the case of a rectovestibular fistula and of an accompanying atretic or absent vagina, two orifices will be found on physical exam—the urethra and fistula. If a vaginal orifice cannot be identified, this should prompt a diagnostic laparoscopy 3 so that the intraabdominal Mullerian anatomy can be ascertained. While this does not need to be done immediately in the neonatal period, it should be done prior to a planned repair. In all cases of rectovestibular fistula, even when a vaginal opening is identified, a careful inspection of the introitus, and even vaginoscopy, is appropriate to be certain that the vaginal anatomy is typical. When anomalies are noted in patients with rectovestibular fistula and patent vaginal openings, the most common finding is a vaginal septum, which also may need to be repaired, either at the time of the anorectal repair or at puberty. 2

There are multiple options for surgical reconstruction when presented with a rectovestibular fistula and an absent or atretic vagina. One strategy is to leave the distal rectum as the neovagina and mobilize the proximal rectum through the anal sphincter to create the neorectum. This minimizes the possibility of injury to the urethra by preventing extensive dissection of the common wall of the urethra and rectum. 4 Also, the distal rectum often lies exactly where the introitus belongs, making this option tempting. The neovagina can be anastomosed to the remnant upper vagina if one exists. Anastomosing it to the uterus if there is no cervix is incorrect as that can lead to ascending infections. In such a rare case, a hysterectomy is warranted. 3

The main problem with leaving the distal rectum to become the neovagina, however, is the loss of the rectal reservoir, which can have negative implications on bowel control. The benefit of using the native rectum in the anorectoplasty is the preservation of the rectal reservoir as well as any inherent sensory or proprioceptive function the distal rectum may provide. 2 This is why it is advised in a patient with good potential for achieving bowel control (a patient with a normal sacrum, good pelvic muscles, and no associated spinal anomaly) to keep the distal rectum as rectum as one would for a typical vestibular fistula case. In this case, since the patient had a normal sacrum with a lateral sacral ratio greater than 0.7 cm, this approach of mobilizing the distal rectum into the sphincter complex was taken to maximize bowel control potential.

When using the native rectum for the anorectoplasty, the separation of the distal rectum from the urethra is needed—this plane is thicker than the one that surgeons are used to when they separate the rectum from the posterior vaginal wall. When a vaginal pull-through is not feasible, vaginal reconstruction can be done with a segment of colon or small bowel. While colon is preferred for use as vaginal replacement due to its abundant blood supply, the ileum has also been used to create a neovagina. 2 3 Another option is to wait to complete the vaginal repair till after puberty, with the potential that the distal atretic vagina will be longer and be more easily mobilized to reach the perineum for a pull-through. If there are no intraabdominal Mullerian structure and the introitus is adequate, the introitus could be dilated later in life. 2 3 4

**Fig. 3:**
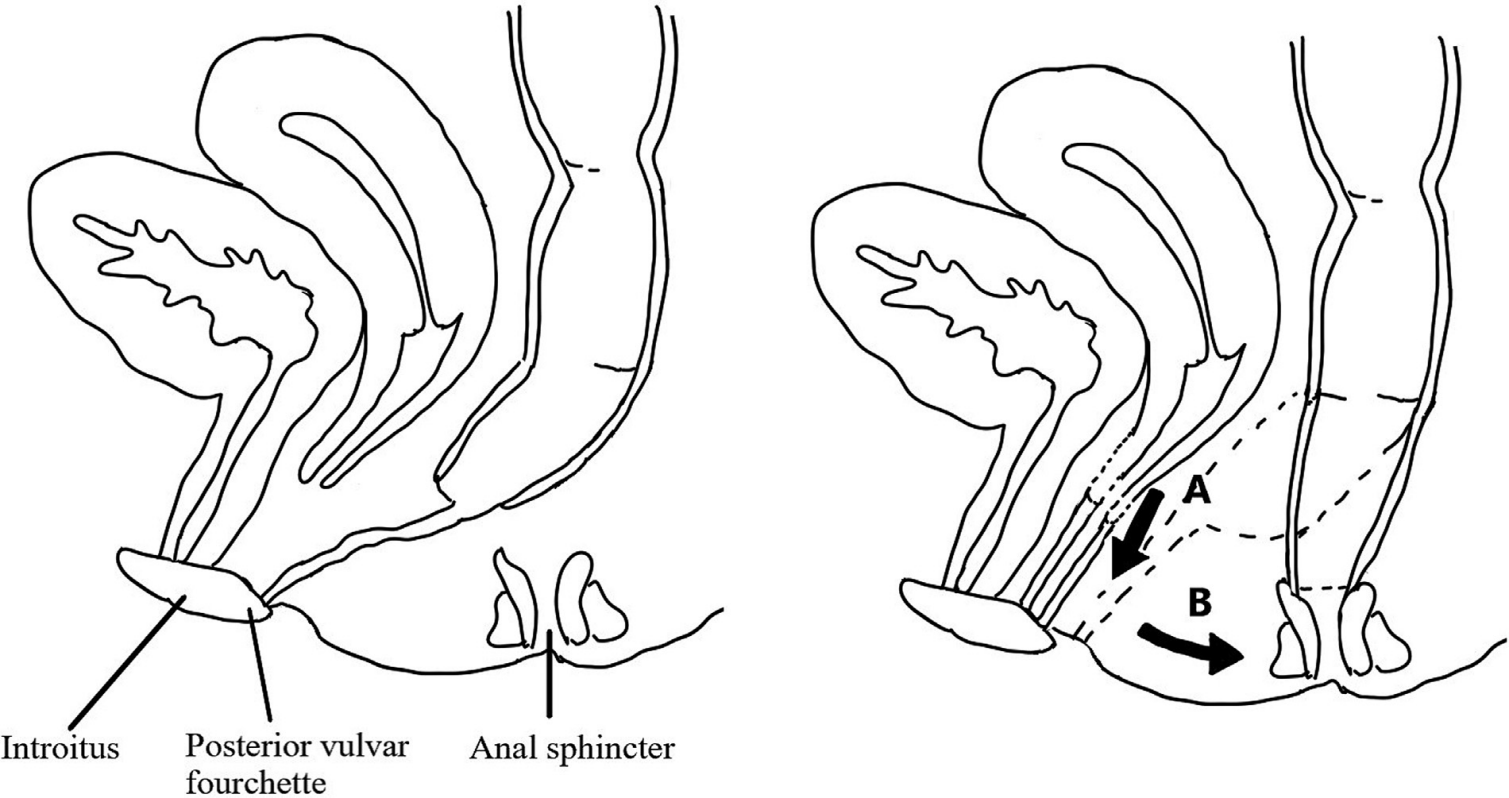
Due to an intact right-sided uterus and vagina, the vagina can be mobilized to the introitus via a pull-through ( **A** ), while the distal rectum is mobilized and reinserted into the anal sphincter through a posterior sagittal anorectoplasty ( **B** ).

Given that the patient in this case has a unicornuate uterus and the presence of proximal vagina on the right side (a rare finding), a vaginal pull-through is a viable option (see Fig. 3 ). While we were prepared to do a bowel neovagina if necessary, the patient's native vaginal reached quite nicely. This approach alleviates the obstruction of Mullerian structures on this side, allowing for menstrual egress and preserving reproductive capacity while also avoiding the need for vaginal replacement. The left atretic and nonpatent Mullerian structures can be excised; however, observation with pelvic ultrasound throughout puberty to ensure there is no accumulation of hematocolpos would also be appropriate if excision is not completed. If the atretic structures are removed, the left Fallopian tube should be removed as well, as this intervention helps reduce the risk of ovarian cancer. 5

Most of the available literature advocates for vaginal repair at the same time as ARM repair, especially if both are noted in the newborn period. This is because the ARM repair involves dissection of the introitus and perineal body, so a future vaginal replacement would be more difficult given accumulation of postoperative scarring. It is important to consider ethical implications of the decisions regarding vaginal replacement in infants—or any patient unable to participate in informed consent—given that vaginal replacement involves alteration of anatomy linked to sexual identity as well as function. Such ethical implications have been routinely evaluated in patients with differences in sexual development, 6 favoring postponement of nonurgent surgical alteration of the genital tract until a patient is capable of, at minimum, assent. While these ethical considerations remain important in patients with ARMs requiring vaginal pull-through or vaginal replacement procedures, it is also important to weigh risks of delay in these procedures. These risks include development of menstrual obstruction at puberty and potential surgical complications associated with performance of vaginal pull-through or replacement procedure after anorectoplasty has been performed. Notably, there are reported cases of vaginal reconstruction done later in life in patients who were not diagnosed with vaginal atresia or agenesis until adolescence, with good outcomes in urinary and stool continence. 7

In addition, such patients need to be specifically evaluated for associated urologic issues as patients with this specific ARM type tend to have associated urologic problems, similar to those found in the MRKH complex. As with all patients born with imperforate anus, it is important to inspect for other anatomic anomalies associated with VACTERL (Vertebral defects, Anal atresia, Cardiac defects, Tracheoesophageal fistula, Renal anomalies, and Limb abnormalities). The patient in this case had no other associated VACTERL or urological anomalies.

## Conclusion

Rectovestibular fistulas are the most common ARM in females. Patients with rectovestibular fistulas may also present with Mullerian abnormalities, and although rare, Mullerian anomalies may include distal vaginal atresia. In these circumstances, the newborn physical exam will reveal two orifices in the perineum and absence of vaginal opening. 2 7 Upon closer inspection, however, a vaginal orifice can be differentiated from a rectal orifice depending on the location of the opening relative to the introitus in the vaginal vestibule. 2 Atypical vaginal anatomy, particularly when a vaginal opening cannot be located, should prompt a diagnostic laparoscopy to delineate gynecologic anatomy prior to ARM primary repair. Early identification of gynecologic anomalies allows for gynecological reconstruction at the same time as the primary ARM repair.

## Quiz Answers

1. C. Meconium is passing through the posterior vulvar fourchette and there is no vaginal opening.
2. C. Rectovestibular fistula with distal vaginal atresia.
3. C. PSARP and vaginal pull-through.

## Notes

New Insights & the Importance for the Pediatric Surgeon We highlight the presence of gynecological anomalies associated with anorectal malformations and the importance of recognizing these anomalies prior to performing definitive surgical repair. We believe readers will find this case report helpful in identifying when to use diagnostic laparoscopy to evaluate for abnormal Mullerian anatomy and how to select the appropriate gynecological and anorectal surgical reconstruction when indicated.
